# Comparisons of Thyroid Hormone, Intelligence, Attention, and Quality of Life in Children with Obstructive Sleep Apnea Hypopnea Syndrome before and after Endoscopic Adenoidectomy

**DOI:** 10.1155/2015/523716

**Published:** 2015-01-15

**Authors:** Hui-Wei Feng, Tao Jiang, Hong-Ping Zhang, Zhe Wang, Hai-Ling Zhang, Hui Zhang, Xue-Mei Chen, Xian-Liang Fan, Yu-Dong Tian, Tao Jia

**Affiliations:** ^1^Department of Otorhinolaryngology, The Second Hospital of Shandong University, Jinan 250000, China; ^2^Department of Otorhinolaryngology, Dezhou Municipal Hospital, Dezhou 253000, China; ^3^Department of Orthopedic Surgery, The First Hospital of Jinan, Jinan 250000, China

## Abstract

*Objective*. The aim of this study was to compare the differences in thyroid hormone, intelligence, attention, and quality of life (QoL) of children with obstructive sleep apnea hypopnea syndrome (OSAHS) before and after endoscopic adenoidectomy. *Method*. A total of 35 OSAHS children (21 males and 14 females with a mean age of 6.81 ± 1.08 years) were included in this study for analyzing the levels of thyroid hormone, intelligence, attention, and QoL. There were 22 children underwent endoscopic adenoidectomy with bilateral tonsillectomy (BT), while the other 13 children who underwent endoscopic adenoidectomy without bilateral tonsillectomy without BT. *Results*. Our results revealed no significant difference in serum free triiodothyronine (FT3), free thyroxine (FT4), and thyroid stimulating hormone (TSH) levels in OSAHS children before and after endoscopic adenoidectomy (all *P* > 0.05). However, there were significant differences in full-scale intelligence quotient (FIQ) (92.45 ± 5.88 versus 106.23 ± 7.39, *P* < 0.001), verbal intelligence quotient (VIQ) (94.17 ± 15.01 versus 103.91 ± 9.74, *P* = 0.006), and performance intelligence quotient (PIQ) (94.12 ± 11.04 versus 104.31 ± 10.05, *P* = 0.001), attention (98.48 ± 8.74 versus 106.87 ± 8.58, *P* < 0.001), and total OSA-18 scores (87.62 ± 17.15 versus 46.61 ± 10.15, *P* < 0.001) between before and after endoscopic adenoidectomy in OSAHS children. *Conclusion*. Our findings provided evidence that the intelligence, attention, and QoL of OSAHS children may be significantly improved after endoscopic adenoidectomy.

## 1. Introduction 

Obstructive sleep apnea hypopnea syndrome (OSAHS) is a respiratory disease caused by total or partial collapse on the upper airway [1]. It is characterized by recurrent airflow obstruction with symptoms and features such as excessive daytime sleepiness or hypersomnia, snoring, morning dry mouth upon awakening, and chest retraction during sleep [2]. With transient nocturnal hypoxia and poor quality sleep, pediatric OSAHS can negatively impact daytime alertness, school performance, behavior, and cardiovascular status and even lead to cognitive delays, hypertension, and heart failure [3]. OSAHS, with its peak incidence between 2 and 8 years of age, can affect 2~3% of children [4, 5]. However, snoring, the main symptom of pediatric OSAHS, is much more frequent ranging from 8% to 27% [6]. In Asia, the prevalence of pediatric OSAHS has ranged from 0% to 4.8% depending on the diagnosis criteria [7]. The etiology of OSAHS is multifactorial which is associated with hypertrophic adenoids, hypertrophic rhinitis, low soft palate, deviations of oral structures, and even hypothyroidism [8]. Comorbidities of OSAHS could be hypertension, insulin resistance, congestive heart failure, stroke, and cerebrovascular ischemic events [9]. Treatment of OSAHS can be divided into three categories, that is, conservative management, alleviation of airway obstruction, and surgery [10]. Lifestyle modification is a kind of conservative management which contains weight reduction, drugs and sedatives withdrawal, enough sleep, and sleeping positions adjustment [10]. As for alleviation of airway obstruction, continuous positive airway pressure and mandibular repositioning splints are preferred [11, 12]. However, surgeries such as tonsillectomy, tracheostomy, jaw advancement surgery, and maxilla-mandibular osteotomy are among the most common treatments of OSAHS with tonsillectomy highly effective in children [13, 14].

Adenotonsillectomy or adenoidectomy, by resecting tonsillar lymphoid tissue to remove the obstruction of the upper airway, is frequently performed for pediatric OSAHS [15]. Great improvements of thyroid function, intelligence, and attention and life quality of OSAHS patients after surgery have been observed by some studies, which can be indicators for tonsillectomy evaluation [16]. As one of the four indicators, the proposed relationship between hypothyroidism and OSAHS could be the deposition of mucoproteins in the upper airway, disturbances of the regulatory control of pharyngeal dilator muscles, and respiratory center depression [17]. And study has showed the recovery of OSAHS symptoms after hypothyroid replacement therapy [18]. Pediatric OSAHS may affect neurocognitive and behavioral domains of children, especially in attentional capacity and intelligence [19, 20]. The disruption in the normal sleep architecture, hypoxemia, and hypercapnia caused by OSAHS could have an effect on prefrontal cortex lesions which play an important role in recognition and identification and finally affect children intelligence and attention [21]. The impact of OSAHS is not only limited to the disease symptoms alone, but also linked to life quality especially in activity limitation, emotions, and interpersonal relationships [22, 23]. Due to the close connection between the four indicators and pediatric OSAHS, the surgery could have a great influence over the four indicators which in return can be the prediction for the valuation of the surgery. Therefore, we performed the present study to compare the differences in thyroid hormone, intelligence, attention, and quality of life (QoL) of children with OSAHS before and after endoscopic adenoidectomy, which could provide practical and reliable evaluation method for clinic diagnosis and treatment of OSAHS.

## 2. Materials and Methods

### 2.1. Ethics Statement

The study design was reviewed and approved by the Ethics Committee of the Second Hospital of Shandong University. The parents or guardians of all children had to sign informed consent in written form to undergo diagnostic and therapeutic procedure at the time of hospitalization.

### 2.2. Patients and Treatment

From September 2010 to June 2011, a total of 35 OSAHS children (21 males and 14 females with a mean age of 6.81 ± 1.08 years) with duration of 6 months to 2 years, and typical symptoms of restless sleep, snoring, apnea, mouth breathing, hyperactivity, sweating, night terrors, night crying, enuresis, daytime sleepiness and so on, were recruited at the Department of Otolaryngology of the Second Hospital of Shandong University. According to the Diagnoses Standard Clinical Practice Guideline: Diagnosis and Management of Childhood Obstructive Sleep Apnea Syndrome (draft) advanced by subcommittee on Chinese Journal of Otorhinolaryngology Head and Neck and Chinese Otolaryngology Research Team in Urumqi in 2007, all recruited children underwent overnight polysomnography for more than 7 hours and were proved to conform to diagnostic criteria. Children with previous otorhinolaryngologic surgery, acute or chronic renal diseases, hematological diseases, congenital heart diseases, inborn error of metabolism, cerebral palsy, and known intellectual disability due to a syndrome or hypothyroidism were not eligible to be enrolled into the study. All children with OSAHS underwent surgery using tracheal intubation under intravenous anaesthesia. Of these children, 22 children underwent bilateral tonsillectomy and endoscopic adenoidectomy, and the other 13 children underwent endoscopic adenoidectomy without bilateral tonsillectomy. All OSAHS children were followed up more than 6 months after surgery. Another 35 healthy children (17 males and 18 females; mean age 6.47 ± 1.51 years) without snore symptom were enrolled randomly as the control group during their visit to our pediatric outpatient clinic for routine physical examination.

### 2.3. Evaluation of Thyroid Hormone Concentration

The serum of OSAHS children was collected preoperatively and postoperatively. Serum free triiodothyronine (FT3), free thyroxine (FT4), and thyroid stimulating hormone (TSH) levels were measured in the morning for 4 weeks using the electrochemiluminescence immunoassay (ECLIA) method (Roche Diagnostics GmbH, Basel, Switzerland) twice, before the treatment and after hormones stabilization. The reference ranges for FT3, FT4, and TSH are 3.0~9.0 pmol/L, 10.3~25.8 pmol/L, and 0.25~5.0 *μ*IU/mL, respectively.

### 2.4. Quality of Life (QoL) Assessment

The disease specific QoL for children with obstructive sleep apnea 18-item survey (OSA-18) was used to measure QoL in children with OSAS and improvement in QoL after the treatment [24]. We used OSA-18 to assess QoL before and 6 months after treatment in five domains, including sleep-disordered breathing, physical symptoms, emotions, daytime behaviors, and impact of guardians. The OSA-18, consisting of 18 relevant items, is scored based on seven levels regarding the frequency of symptoms: (1) never: 1; (2) hardly: 2; (3) seldom: 3; (4) sometimes: 4; (5) frequently: 5; (6) mostly: 6; (7) absolutely: 7. Total OSA-18 scores range from 18 (lowest) to 126 (highest). According to the OSA-18 scores, the included subjects were classified into three levels: mild OSAS (<60), moderate OSAS (60–80), and sever OSAS (>80), respectively. In addition, the improvement in QoL after the treatment was assessed with change in OSA-18 scores, which was measured by the differences of preoperative average OSA-18 scores (OSA-18 total score/18) with postoperative average OSA-18 scores, ranging from −7.0 to +7.0. A negative value indicates deterioration, while a positive value indicates improvement, which was further divided into 4 degrees: (1) <0.5, slight improvement; (2) 0.5–0.99, mild improvement; (3) 1.00–1.49, moderate improvement; and (4) ≥1.5, obvious improvement.

### 2.5. Cognition and Attention Assessment

General intellectual functioning was assessed by a gold standard instrument, the Chinese Wechsler Intelligence Scale for Children (C-WISC), which is applicable for children ranging in age from 6 to 16 years. All tests were performed in a separated and quiet room by a trained psychiatrist. The C-WISC is composed of 11 distinct subtests divided into two scales, a verbal scale (VS) and a performance scale (PS). The VS includes information (I), comprehension (C), picture generalization (PG), arithmetic (A), and picture and vocabulary (PV); and PS consists of picture arrangement (PA), picture completion (PC), block design (BD), animal egg (AE), maze analysis (MA), geometric design (GD), and vision analysis (VA). The verbal intelligence quotient (VIQ) was measured by VS that are language based items, performance intelligence quotient (PIQ) was measured by PS that are visual motor items and are less dependent on language, and full intelligence quotient (FIQ) was measured by full scale (FS). Before and 6 months after treatment, eleven of the subtests in each scale were assessed to produce scale-specific intelligence quotients (IQs) such as VIQ, PIQ, and FIQ (range from 85 to 115 in normal subjects), as well as concentration factor (factor C).

### 2.6. Statistical Analysis

Data are presented as mean ± standard deviation. The* t*-test was applied in the comparison of thyroid hormone, intelligence, attention, and QoL between before and after endoscopic adenoidectomy. Further comparison among the three groups was performed using the analysis of variance (ANOVA). All statistical analyses were conducted using the SPSS 17.0 software (SPSS Inc., Chicago, Illinois, USA). A *P* value of < 0.05 was considered statistically significant.

## 3. Results 

### 3.1. Baseline Characteristics

There was no significant difference in age, gender, body mass index (BMI), height, and weight between OSAHS and healthy children (all *P* > 0.05). However, significant differences were observed in osteocalcin (7.41 ± 1.31 versus 10.22 ± 2.50, *P* = 0.037) and the bone age (−0.51 ± 0.21 versus −0.02 ± 0.01, *P* = 0.038). The baseline characteristics of the individual subject were presented in Table 1.

### 3.2. Changes of Thyroid Hormone, Intelligence, Attention, and Quality of Life before and after Endoscopic Adenoidectomy

As shown in Table 2, there were no significant differences in serum FT3, FT4, and TSH levels in OSAHS children before and after endoscopic adenoidectomy (all *P* > 0.05). However, there were significant differences in FIQ (92.45 ± 5.88 versus 106.23 ± 7.39, *P* < 0.001), VIQ (94.17 ± 15.01 versus 103.91 ± 9.74, *P* = 0.006), and PIQ (94.12 ± 11.04 versus 104.31 ± 10.05, *P* = 0.001), attention (98.48 ± 8.74 versus 106.87 ± 8.58, *P* < 0.001), and total OSA-18 scores (87.62 ± 17.15 versus 46.61 ± 10.15, *P* < 0.001) between before and after endoscopic adenoidectomy in OSAHS children. Furthermore, we also observed differences in thyroid hormone, intelligence, attention, and QoL between OSAHS children before surgery and healthy children (all *P* < 0.05).

### 3.3. Comparisons of Thyroid Hormone, Intelligence, Attention, and Quality of Life between OSAHS Children with and without Bilateral Tonsillectomy

The present study demonstrated that OSAHS children with BT had higher FIQ, VIQ, and PIQ and lower OSA-18 scores than those of OSAHS children without BT (all *P* < 0.05). Nevertheless, we also found no significant differences in serum FT3, FT4, and TSH levels between OSAHS children with BT and those without BT (all *P* > 0.05).

## 4. Discussion

In this present study, we aimed to explore the differences in thyroid hormone, intelligence, attention, and QoL of children with obstructive sleep apnea hypopnea syndrome (OSAHS) before and after endoscopic adenoidectomy. The findings in our study have revealed that the intelligence, attention, and QoL in children with OSAHS after surgery were significantly improved compared with those before surgery, suggesting that the intelligence, attention, and QoL of OSAHS children may be significantly improved after endoscopic adenoidectomy. It has been well known that OSAHS may result in various kinds of pathophysiological consequence including hypertension, functional impairments of heart, polycythemia, and lesions in many other systems and when present it may contribute to worse outcomes in human bodies [25]. Owing to these symptoms and functional impairments, patients with OSAHS, especially children, usually suffer from cognitive impairment and have a poor QoL from physical and emotional health to social functioning [26]. Children with OSAHS may experience several neurobehavioral problems, such as attention deficit, learning problems, memory deficits, hyperactive, behavioral disorders, and hypersomnolence, and it has also been linked to lower childhood IQ scores [27]. Many researchers have claimed that sleep-related breathing disorders including OSAHS, high microarousal index, intermittent hypoxia, and alterations in the sleep architecture may result in insufficient growth hormone, impairment of cognitive function, abnormalities of behavior and psychology, and so on [27, 28]. Among children, these complications are frequently noted clinically as decreased memory and learning abilities, aprosexia, hypomnesia, impairment of memory function, mania, and depression [29]. Moreover, OSAHS is often associated with oxygen deficit and metabolic disturbance which may result in cognitive impairment, abnormalities of behavior and psychology [26]. A previous study performed by Yang et al. has revealed that intermittent hypoxia resulting from OSAHS may cause structural neuron damage and dysfunction in the central nervous system, especially in the hippocampus, which can be regarded as potential mechanisms of neurocognitive and behavioral deficits [30].

The main finding of this study demonstrates that the scores of FIQ, VIQ, and PIQ in OSAHS children were obviously decreased after the operation when compared with the scores in preoperative children with OSAHS, suggesting that operation can improve the response control, attention, and hyperactivity behaviors of children with OSAHS. In addition, the continuous performance test revealed that factor C was significantly increased in the patients with OSAHS after operation in comparison with those before operation, implying that the early surgical treatment for OSAHS can improve the symptom of cognitive impairment and enhance attention of children. As an attention/vigilance factor, factor C can reflect some characteristics of attention impairment and closely relates to the school performance in children [19, 31]. Thus, the increased factor C may indicate that surgical treatment of OSAHS children can promote the recoveries of attention impairment and further improve the school performance of children. As stated above, children with OSAHS often suffer from obviously decreasing cognitive function including lower learning abilities, decreased memory performance, and aprosexia, which may largely influence the QoL; thus children with OSAHS frequently have poor QoL. In this regard, we suspected that the improvement and recovery of intelligence and attention in OSAHS children may lead to the improvement of QoL. In this study, we adopted OSA-18 to assess the QoL in children with OSAHS from five parts including sleep-disordered breathing, physical symptoms, emotions, daytime behaviors, and impact of guardians [24]. Our results have revealed that the total OSA-18 scores after operation were significantly decreased compared with the preoperative total OSA-18 scores in children with OSAHS, suggesting that the QoL in children may be affected by OSAHS, and the surgical treatment can improve the QoL in children.

Thyroid hormone is primarily responsible for regulation of metabolism, which can increase the basal metabolic rate, affect protein synthesis, and regulate long bone growth (cooperate with growth hormone) and neural maturation [32, 33]. Also, it has been reported that the thyroid hormone plays crucial roles in the cells proliferation and differentiation in human body, and various physiological and pathological stimuli may influence thyroid hormone synthesis [34, 35]. However, a study performed by Lanfranco F has demonstrated that obese patients with OSAHS were not obviously related to impaired thyroid activity in either basal conditions or an altered TSH response to TRH challenge test [36]. In consistent with previous study and document, our study also found that there was no significant difference between the serum levels of T4, FT3, and TSH in OSAHS children in pretreatment and posttreatment periods. The possible reason for this outcome may be that the severity of intermittent hypoxia caused by OSAHS cannot induce the decreased secretion of thyroid hormone. One limitation of the studies that assessed the changes of serum levels of T4, FT3, and TSH in OSAHS children is that small sample size in our study and all OSAHS children were not well grouped. We need to conduct further studies to explore the relationship between the severe OSAHS and thyroid function in children.

In summary, our findings provided evidence that the intelligence, attention, and QoL of OSAHS children may be significantly improved after endoscopic adenoidectomy. However, due to many limitations, our conclusion should be confirmed in a larger prospective trial.

## Figures and Tables

**Table 1:** Baseline characteristics of children with obstructive sleep apnea hypopnea syndrome (OSAHS) and healthy children.

Characteristic	OSAHS group (*n* = 35)	Control group (*n* = 35)	*χ* ^2^/*t*	*P* value
Age (years)	6.81 ± 1.08	6.47 ± 1.51	0.214	0.832
Gender (male/female)	21/14	17/18	0.921	0.337
Body mass index (BMI)	22.58 ± 3.22	22.49 ± 2.89	0.897	0.372
Height (cm)	1.06 ± 0.20	1.07 ± 0.27	1.470	0.293
Weight (kg)	25.41 ± 4.36	25.70 ± 3.55	0.325	0.747
Bone age	−0.51 ± 0.21	−0.02 ± 0.01	1.789	0.038
Osteocalcin (*μ*g/L)	7.41 ± 1.31	10.22 ± 2.50	2.142	0.037

**Table 2:** Thyroid hormone, intelligence, attention, and quality of life of OSAHS children before and after endoscopic adenoidectomy and healthy children.

Group	*N*	FT_3_ (*μ*mol/L)	FT_4_ (*μ*mol/L)	TSH (uIU/mL)	FIQ	VIQ	PIQ	OSA-18
*OSAHS group *								
Before surgery	35	6.04 ± 1.88	14.17 ± 5.01	4.12 ± 1.04	92.45 ± 5.88	94.17 ± 15.01	94.12 ± 11.04	87.62 ± 17.15
After surgery	26	6.23 ± 2.39	13.91 ± 4.74	4.31 ± 1.05	106.23 ± 7.39^*^	103.91 ± 9.74^*^	104.31 ± 10.05^*^	46.61 ± 10.15^*^
With BT	22	6.46 ± 1.73	12.81 ± 3.26	4.07 ± 0.88	112.05 ± 6.87^*^	109.22 ± 8.18^*^	109.54 ± 9.97^*^	39.89 ± 11.23^*^
Without BT	13	6.12 ± 3.06	14.09 ± 4.91	4.67 ± 1.32	103.14 ± 8.13^*^	98.77 ± 10.27	101.28 ± 11.83^*^	51.24 ± 9.64^*^
*Control group *	35	6.21 ± 2.45	14.21 ± 4.63	4.33 ± 1.29	110.59 ± 8.32^*^	106.91 ± 11.25^*^	109.37 ± 7.91^*^	37.81 ± 8.67^*^

FT_3_: free triiodothyronine; FT_4_: free thyroxine; TSH: thyroid stimulating hormone; FIQ: full-scale intelligence quotient; VIQ: verbal intelligence quotient; PIQ: performance intelligence quotient; ^*^ *P* < 0.05 compared with the levels before surgery.
